# Gallbladder adenosquamous carcinoma: a case report and a review of the role of laparoscopy in managing a rare entity

**DOI:** 10.1093/jscr/rjaf805

**Published:** 2025-12-28

**Authors:** Hadar Yafee, Yaron Rudnicki, Sagi Tal, Guy Lifshitz, Shmuel Avital, Lauren Lahav

**Affiliations:** Department of Surgery B, Meir Medical Center, Affiliated with Faculty of Medical & Health Sciences, Tel Aviv University, Tel Aviv, Israel; Department of Surgery B, Meir Medical Center, Affiliated with Faculty of Medical & Health Sciences, Tel Aviv University, Tel Aviv, Israel; Department of Surgery B, Meir Medical Center, Affiliated with Faculty of Medical & Health Sciences, Tel Aviv University, Tel Aviv, Israel; Department of Surgery B, Meir Medical Center, Affiliated with Faculty of Medical & Health Sciences, Tel Aviv University, Tel Aviv, Israel; Department of Surgery B, Meir Medical Center, Affiliated with Faculty of Medical & Health Sciences, Tel Aviv University, Tel Aviv, Israel; Department of Surgery B, Meir Medical Center, Affiliated with Faculty of Medical & Health Sciences, Tel Aviv University, Tel Aviv, Israel

**Keywords:** adenosquamous carcinoma, gallbladder malignancy, radical cholecystectomy, diagnostic laparoscopy

## Abstract

Gallbladder adenosquamous carcinoma is a rare, aggressive malignancy (1%–10% of gallbladder cancers), with National Comprehensive Cancer Network guidelines not differentiating between histological subtypes. Radical cholecystectomy remains the only curative treatment, though laparoscopic feasibility is debated. We present a woman in her 50s with abdominal discomfort and imaging showing gallbladder wall thickening and an “hourglass appearance”. Diagnostic laparoscopy revealed a firm mass without metastasis. Following laparoscopic cholecystectomy, intraoperative pathology confirmed adenocarcinoma, prompting immediate radical cholecystectomy, initiated laparoscopically, completed via open approach. Pathology demonstrated a 2.7 cm moderately differentiated adenosquamous carcinoma with negative margins and uninvolved lymph nodes (pT2aN0). Adjuvant chemotherapy was administered, and the patient was disease-free at one year follow-up. Documenting such rare cases is essential for advancing disease understanding. This case underscores the potential for favourable prognosis with early detection and highlights the value of integrating laparoscopic techniques in a multidisciplinary approach to optimize surgical outcomes while balancing safety and recovery.

## Introduction

Gallbladder carcinoma is a relatively rare malignancy, with a global incidence ranging between 1 and 3 cases per 100 000 individuals annually [1]. Adenosquamous carcinoma (ASC), a rare histological subtype, accounts for ~1%–10% of all gallbladder cancers, rendering it a particularly uncommon entity [2–4]. As a result, the available literature is limited, composed predominantly of isolated case reports and clinicopathological reviews.

ASC demonstrates a predilection for females and shares common risk factors with other subtypes, most notably gallstones [1, 2, 4, 5]. It is associated with more aggressive biological behaviour, reflected in higher rates of lymph node involvement and frequent invasion into adjacent hepatic tissue [1, 2]. Compared to the more prevalent adenocarcinoma, ASC presents with a poorer prognosis, with 5-year overall survival ranging from 8% to 20% and median survival typically under 6 months [2–4, 6, 7].

Histologically, ASC features both glandular elements, which secrete mucin, and squamous components, which confer increased local invasiveness. Tumours exhibiting >25% squamous differentiation meet the criteria for this subtype [8]. The pathogenesis remains uncertain, but two main theories predominate: the squamous metaplasia theory suggests chronic irritation—such as that induced by gallstones—promotes squamous transformation of glandular epithelium, whereas the differentiation theory posits that the tumour arises via transdifferentiation from pre-existing adenocarcinoma. Molecular studies have demonstrated shared genetic mutations in the glandular and squamous components, supporting the latter hypothesis [4, 9].

The glandular component is more likely to metastasize distantly, while the squamous element contributes to local aggressiveness due to its high proliferative index and capacity to infiltrate surrounding tissues [3, 4, 10]. The liver is most commonly invaded, followed by adjacent gastrointestinal structures. Surgical resection and adjuvant systemic therapy are consistently associated with improved prognosis [2, 3, 6, 7]. Herein, we present a case of a woman diagnosed intraoperatively with ASC of the gallbladder, managed successfully using a combination of laparoscopic and open surgical techniques, followed by adjuvant chemotherapy.

## Case report

A 55-year-old female presented to her primary care physician with right-sided abdominal and flank discomfort persisting for over a month. Her medical history included ulcerative colitis managed with mesalamine and biopsy-confirmed nonalcoholic fatty liver disease diagnosed 2.5 years earlier. She had been undergoing gastroenterological follow-up due to persistently elevated liver enzymes.

**Figure 1:**
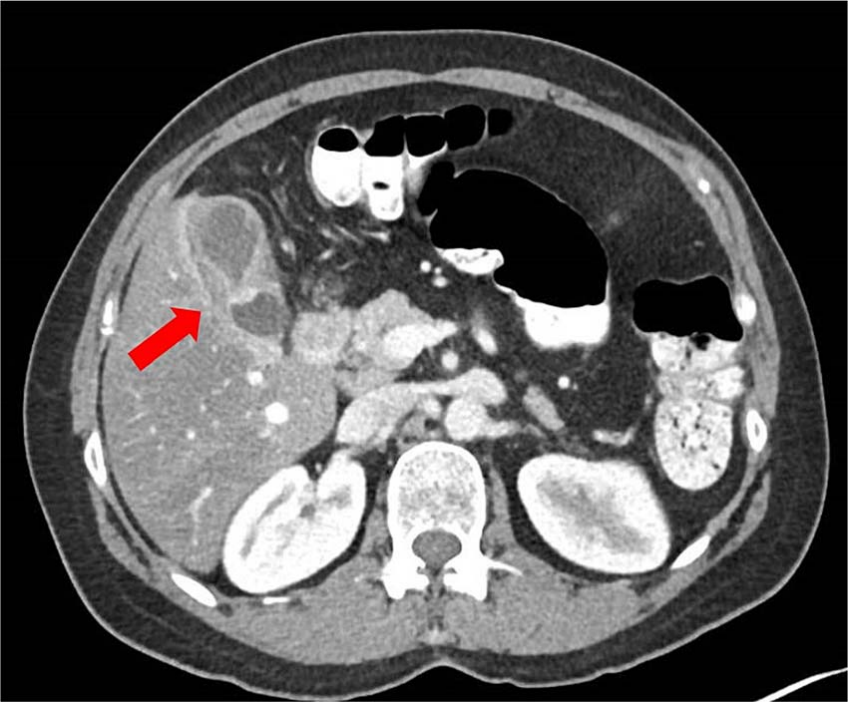
Abdominal CT scan showing an ‘hourglass’ appearance of the gallbladder (arrow).

Initial abdominal ultrasonography was inconclusive. A follow-up fasting ultrasound revealed vascular, thickened content within the gallbladder, suggestive of either cholelithiasis or malignancy. The liver appeared moderately fatty without enlargement or biliary ductal dilatation. A contrast-enhanced computed tomography (CT) scan demonstrated gallbladder wall thickening and a luminal constriction at mid-body, producing a classic “hourglass” appearance (Fig. 1).

The patient was referred for surgical consultation. Physical examination was unremarkable, with no icterus or palpable mass. Laboratory results revealed mild but stable transaminase elevations over several years (aspartate aminotransferase 40 U/L, alanine aminotransferase 50 U/L, gamma-glutamyl transferase 50 U/L), with normal alkaline phosphatase and bilirubin levels. Tumour marker evaluation revealed a notably elevated carcinoembryonic antigen level of 21.4 ng/ml, while carbohydrate antigen 19–9 (CA 19–9) remained within normal range.

Following a multidisciplinary review, the consensus was to proceed with laparoscopic cholecystectomy with the option for immediate conversion to radical resection based on intraoperative findings. On 19 December 2023, laparoscopic cholecystectomy was performed. Intraoperatively, a firm mass adherent to the omentum was identified, without evidence of ascites or peritoneal metastasis. The gallbladder was resected antegradely and submitted for intraoperative frozen section, which confirmed adenocarcinoma.

In light of the pathology findings, conversion to an open radical cholecystectomy was performed, including hepatic non-anatomic (wedge) resection of segments IVb and V and regional lymphadenectomy. Intraoperative ultrasound revealed no hepatic metastases. The procedure was completed without complications, and a Jackson-Pratt drain was placed in the liver bed. The postoperative course was uneventful, and the patient was discharged on postoperative day five.

**Figure 2:**
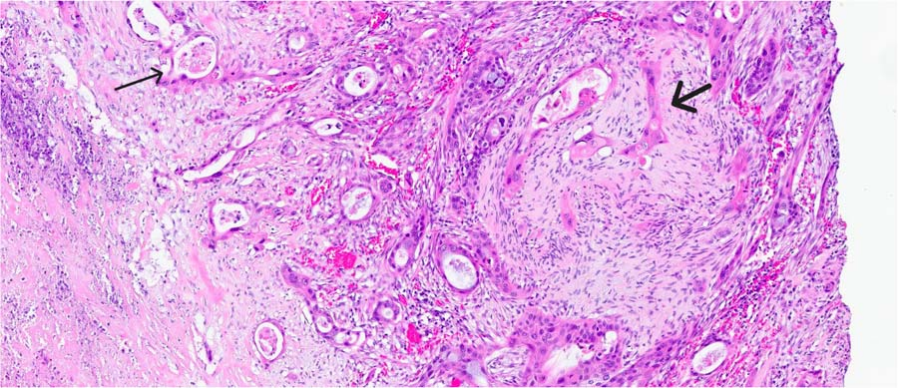
Magnified view (haematoxylin and eosin stain, ×10) of the gallbladder showing mucin glands related to the adenomatous component (narrow arrow) and solid features including stromal infiltration of the squamous components (thick arrow).

**Figure 3:**
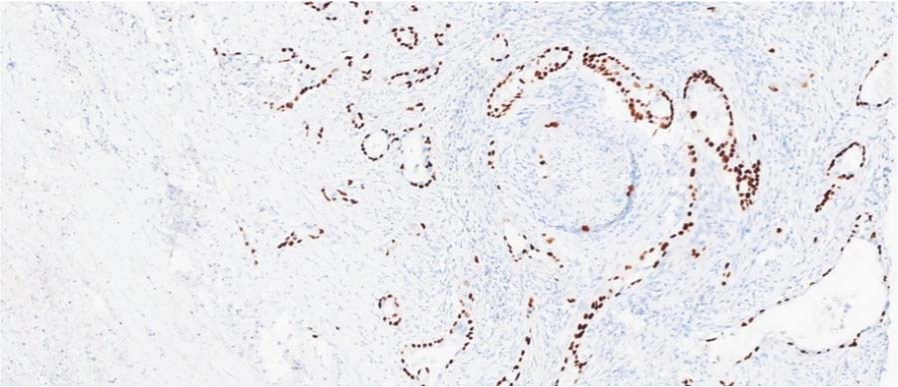
Immunohistochemical stain is positive for p40, highlighting squamous differentiation.

Histological evaluation revealed a 2.7 cm moderately differentiated ASC with extensive necrosis (Fig. 2), perimuscular connective tissue invasion, and lymphovascular involvement. Immunohistochemistry was positive for p40, confirming squamous differentiation (Fig. 3). All surgical margins were uninvolved, and three examined lymph nodes were negative. The tumour was staged as pT2aN0 based on the American Joint Committee on Cancer (AJCC) 8th edition criteria [11].

One month later, a positron emission tomography (PET)-CT scan showed a 3.2 cm hypermetabolic collection at the surgical bed, with additional uptake in the portocaval region and abdominal wall—findings attributed to postoperative inflammatory changes. Two months post-surgery, the patient began adjuvant chemotherapy with Capecitabine (Xeloda) and Oxaliplatin. After six cycles, an allergic reaction necessitated discontinuation of Oxaliplatin. She continued on Capecitabine for a total of ten months.

Follow-up ultrasonography revealed no pathological findings beyond pre-existing hepatic steatosis. A repeat PET-CT demonstrated complete resolution of hypermetabolic activity. At one-year follow-up, the patient remained disease-free, with only mild neuropathy and fatigue related to chemotherapy.

## Discussion

ASC of the gallbladder is a biologically aggressive and rarely encountered malignancy. The available data are sparse and largely anecdotal, emphasizing the importance of documenting individual cases to contribute to the broader understanding of its behaviour and management.

This case highlights several key issues in diagnosis and treatment. First, the patient presented with non-specific abdominal discomfort, a symptom not typically associated with early-stage gallbladder carcinoma [1, 3]. Nonetheless, appropriate imaging and clinical suspicion facilitated early diagnosis. Importantly, laparoscopic cholecystectomy served as a diagnostic tool, with intraoperative frozen section enabling immediate pathological confirmation and escalation to curative surgery.

According to the National Comprehensive Cancer Network (NCCN) guidelines [12], a suspicious gallbladder mass warrants definitive surgical resection without preoperative biopsy. While staging with multiphasic cross-sectional imaging is typically advised, in this case, direct surgical intervention was chosen to avoid treatment delays. Diagnostic laparoscopy is recommended prior to resection to assess operability. In patients where malignancy is uncertain, performing a cholecystectomy with intraoperative pathology and proceeding with radical surgery if cancer is confirmed is considered acceptable and was implemented here.

The surgical principle followed NCCN guidelines, recommending resection of segments IVb and V with lymphadenectomy. Bile duct excision is added if involvement is suspected. Guidelines do not specify whether resection should be anatomic or wedge, nor differentiate by histologic subtype. In our case, a wedge resection achieved clear margins and no recurrence supporting its oncologic adequacy in T2a ASC of the gallbladder.

Historically, open surgery was preferred for radical cholecystectomy in gallbladder cancer due to concerns about port-site metastases and lymphadenectomy adequacy. Since 2010, studies from high-volume centers show that laparoscopic resection in selected T2 cases achieves comparable outcomes including R0 resection rates, lymph node yield, and long-term survival while reducing blood loss, postoperative complications, and hospital stay [13]. Recent data on robotic approaches show similar results, further supporting minimally invasive surgery as a safe, effective alternative [14].

Several studies provide insight into prognosis and surgical outcomes. Oohashi et al. reported improved five-year survival (48.6%) in patients undergoing radical resection versus limited surgery (7.7%) among a cohort of 29 patients with advanced disease [10]. Similarly, Chan et al. found that curative resection significantly prolonged survival in a cohort of 12 patients [3]. Data from Akce et al., using the U.S. National Cancer Database, indicated that 88.9% of patients with ASC underwent surgery, with surgical resection emerging as a favorable prognostic factor in multivariate analysis [6].

Murimwa et al. later confirmed these findings in a larger dataset, showing that curative-intent resection conferred a 5-year survival of 18% for ASC, compared to 25% for adenocarcinoma. They also identified tumour size >4 cm, nodal positivity, and margin involvement as independent predictors of worse outcomes [7].

Regarding adjuvant therapy, NCCN guidelines recommend Capecitabine following resection for gallbladder carcinoma, with no subtype-specific differentiation. Akce et al. demonstrated that adjuvant systemic therapy—comprising chemotherapy and other modalities—improved survival in patients with ASC, whereas radiation therapy alone had no significant impact [6].

European Society for Medical Oncology guidelines similarly endorses radical surgery and adjuvant Capecitabine. Radiotherapy may be considered in R1 resections [5]. Fang et al. recently found that systemic therapy improved survival in advanced-stage ASC but not in early-stage disease, though treatment details were limited [15].

Our patient, staged as IIA based on the American Joint Committee on Cancer (AJCC) 8th edition criteria, received guideline-directed therapy and remained recurrence-free at 1 year [11]. This outcome highlights the importance of early detection, accurate staging, and a multidisciplinary approach.

In conclusion, ASC of the gallbladder is a rare and aggressive histological subtype with poor prognosis. Despite its distinct biological characteristics, current management strategies do not vary by histology. This case underscores the utility of laparoscopic techniques in diagnosis and staging, enabling timely transition to curative surgery. Comprehensive surgical resection followed by adjuvant chemotherapy remains the cornerstone of treatment. Continued case reporting and research are essential to improve outcomes and refine management for this rare malignancy.
